# The Use of Mycelial Fungi to Test the Fungal Resistance of Polymeric Materials

**DOI:** 10.3390/microorganisms11020251

**Published:** 2023-01-19

**Authors:** Natalya Ivanushkina, Kristine Aleksanyan, Svetlana Rogovina, Galina Kochkina

**Affiliations:** 1 All-Russian Collection of Microorganisms (VKM), Pushchino Scientific Center for Biological Research of the Russian Academy of Sciences, G.K. Skryabin Institute of Biochemistry and Physiology of Microorganisms, Russian Academy of Sciences, 142290 Pushchino, Russia; 2 Semenov Federal Research Center for Chemical Physics, Department of Polymers and Composite Materials, Russian Academy of Sciences,119991 Moscow, Russia; 3 Engineering Center, Plekhanov Russian University of Economics, 117997 Moscow, Russia

**Keywords:** fungi, biodestruction, polymer materials, test-cultures

## Abstract

There are two main themes in the research on the biodegradation of industrial materials by mycelial fungi. The challenge of reducing environmental pollution necessitates the creation of biodegradable polymers that allow microorganisms, including mycelial fungi, to degrade them to low-molecule soluble substances. Additionally, to minimize the biodegradation of industrial materials while they are operating in the environment, there is a need to produce fungi-resistant polymer compositions. The fungal resistance of industrial materials and products can be assessed using a specific set of mycelial fungi cultures. Test cultures selected for this purpose are supported in the All-Russian Collection of Microorganisms (VKM). This review addresses the principle of culture selection to assess the fungal resistance of industrial materials and evaluates the results of the tests using these cultures.

## 1. Introduction

Issues related to testing of the ability of microorganisms to damage industrial materials, both natural and artificial, are important and relevant not only for industrial biotechnology, but also for industry as a whole. At present, it is virtually impossible to put into operation a new industrial sample or product without appropriate testing.

Mycelial fungi are organisms that live everywhere: in the soil, air, and water, and on various materials. They are capable of causing significant damage to building structures and industrial materials [1,2]. Their destructive activities are facilitated by a well-developed enzyme system, excellent adaptive capacities, and a high growth rate [3]. These factors increase the ability of the fungi to adapt quickly to changes in the environment. The mycelial structure and the method of obtaining nutrition by absorbing solutions of organic substances allow them to rapidly colonise almost any surface. Using a rich and diverse set of constitutive and adaptive enzymes, fungi can degrade high-molecular compounds, including synthetic ones [4]. The production of organic acids also increases their destructive activity [5,6].

Direct biodegradation of industrial materials by fungi occurs when they themselves are a nutrient medium for micromycetes. If the nutrients required for fungal growth cannot be extracted from the industrial materials, they can come from the various contaminants that accumulate on the surface during the production, storage, and use of the materials. Further growth of the fungi can lead to the release of metabolic products. These substances can cause damage to industrial materials.

Micromycetes are capable of damaging almost all types of natural and synthetic materials [7,8]. However, the biodamage processes occur at different speeds, which depends on both the specifics of the materials and the aggressiveness of the fungi (biodegrading activity), and are able to develop in negative environmental conditions. Glass was, for a long period of time, thought to be an inert material capable of withstanding the influence of fungi. However, even under extreme conditions, some organisms can cause mechanical damage, reducing the optical properties and breaking the protective top layer of glass lenses [9]. Species of fungi that can develop at an extremely low humidity (less than 50%), such as *Aspergillus tonophilus* and *A. penicillioides*, are examples of fungi that can affect glass [10].

In order to assess which fungi should be used to check the resistance of a given material, it is necessary to find which fungal species are the most common and the most active in the degradation of polymers, fuels, synthetic fibres, etc. In turn, evaluation of the resistance of new industrial materials requires a defined set of fungal test cultures. An analysis of published articles and our own experimental experience in the estimation of fungal resistance of industrial materials and in studying the degradation of newly developed biodegradable polymers allowed us to reach the conclusion that both activities require the use of a specific set of test cultures of mycelial fungi to compare the results of different research and development activities.

## 2. Degradation of Some Industrial Materials by Fungi

Oil hydrocarbons are the main environmental pollutants from manufacturing plants, transport, shipping, and accidental oil spills. Numerous studies have shown that many polycyclic aromatic hydrocarbons are toxic to the environment. They can be biotoxic, phototoxic, genotoxic, and immunotoxic [11]. According to the IARC (International Agency for Research on Cancer), the first group of hazards (carcinogenic to humans) includes primarily benzopyrene, which is considered the most carcinogenic substance of this type. This group also includes engine exhaust, diesel, and mineral oils. Naphthalene and anthracene belong to Group 2B (possibly carcinogenic to humans) (https://monographs.iarc.who.int/list-of-classifications/, accessed on 27 December 2022.). Cleaning of the environment of such PAHs is a major issue, and fungi can play a part in this process.

Fungi affecting fuels, oils, and lubricants are well-known [12,13]. They mainly belong to *Aspergillus*, *Penicillium*, *Fusarium*, *Hormoconis*, *Neosartorya*, *Paecilomyces*, *Talaromyces*, *Graphium*, and *Cunninghamella* genera and are known for their ability to decompose crude oil and its components [14,15]. In particular, *Cunninghamella elegans* has demonstrated the ability to degrade fluoranthene [16], and *Aspergillus terreus* can degrade naphthalene and anthracene [17]. The strain *Penicillium* sp., isolated from Antarctic soil, can decompose acenaphthene at low temperatures [18].

Some of these fungi are used for bioremediation of the environment following polycyclic hydrocarbon pollution. Mycoremediation is becoming a popular bioremediation technique [4]. For example, it has been shown that fungi of the *Aspergillus* and *Fusarium* taxa isolated from the soil in an oil production area can be effectively used in bioaugmentation to remove hydrocarbon pollutants from oilfield wastewater in oil field areas [19]. Environmental factors (the mechanical and chemical composition of soil, temperature, and pH) influencing the bioremediation process with fungi are being actively studied [20].

However, there is a downside to these properties, as the same fungi can inflict damage to human economic activities by degrading the fuel used in many industries [21,22]. 

*Aspergillus fumigatus* is often isolated from aviation fuel and can be active destructors of petroleum products. In this environment, fungal spores are able to survive at temperatures of up to 80 °C [23]. *Hormoconis resinae* (syn. *Cladosporium resinae*, *Amorphotheca resinae*) has a very high decomposition potential for aviation fuel; it has been shown to cause a loss of 17.8–66.9% of base fuel mass in a 14-day incubation period at room temperature [24]. This microorganism, containing dark melanin pigment, is called a ‘kerosene fungus’. Another ‘kerosene fungus’ is *Monascus floridanus*, and is also capable of active growth in aviation fuel [25].

Spores of ‘kerosene fungi’ adapt well to extreme conditions, and they are smaller than the pores of fuel filters. When injected into the fuel tank, they germinate and produce a mycelium which damages the fuel system [26]. The activity of these fungi leads to damage to the pipelines and fuel storage tanks, as well as inefficient operation of aircraft engines [27,28].

It has been shown that biodegradation of fuel occurs due to microbial enzymatic oxidation of hydrocarbons, with the formation of organic acids with surface-active properties. For example, a system of extracellular enzymes of several *Aspergillus* species isolated from soils contaminated with crude oil were shown to effectively decompose hydrocarbons [14]. Aliphatic hydrocarbons are less biostable than aromatic hydrocarbons, and undergo oxidation more rapidly [29].

Fungal damage to paint coatings is another common case of biodegradation [30,31]. Various materials are used to produce such coatings, which differ in composition and chemical properties of the film-forming formers: bitumen and ether-cellulose varnishes; polyester and polyurethane varnishes; drying oils, oil varnishes and paints; and alkyd varnishes.

If the packaging of varnishes and paints is broken, predominantly waterborne materials are mainly affected by microorganisms. Once applied to products, virtually all paint coatings are subject to biodegradation by fungi. The biologically active substances produced by fungi can break down polyurethanes, converting plasticizers into different polymers, resulting in a loss of coating strength. This is detrimental to polymeric lacquers, as the coating becomes more permeable when exposed to the fungi, making it ineffective as a protective layer [32]. Mycological studies have shown that the fungi of the genera *Aureobasidium*, *Alternaria*, *Aspergillus*, *Cladosporium*, and *Penicillium* are the main destroyers of paint coatings [33,34].

The decomposition by micromycetes affects polymeric materials of various chemical compositions and structures, including synthetic plastics. The final disposal of these materials using chemical and physical methods is very expensive, and leads to the formation of persistent organic pollutants. Among these pollutants, furans and dioxins are known to be toxic products that are harmful to animals, humans, and ecosystems in general [35,36]. Therefore, the issue of microbial degradation of artificial polymers has been given very high priority in research. Biodegradation is also an environmentally friendly process that may contribute to solving the problem of plastic waste. 

Extensive research has been carried out to determine the species composition of mycelial fungi capable of destroying polymeric materials of different compositions in nature. According to A.Y. Lugauskas and co-authors [37], there are more than 400 species of fungi known to destroy polymers, including *Aspergillus amstelodami* sensu lato, *A. awamori*, *A. oryzae*, *Chaetomim globosum*, *Paecilomyces variotii*, *Penicillium cyclopium*, *P. chrysogenum*, *Scopulariopsis brevicaulis*, *Trichoderma virens*, and *T. viride*. 

By changing the conditions of the experiment, it is possible to determine how the degree of biodegradation is affected by material composition and environmental conditions. In addition, it is possible to establish which fungal taxa are the most common in the degradation of polymer composites. For example, in the humid marine climate, among the biodegradable fungi of polyethyracrylate polymer composites, the highest frequency of occurrence was recorded in the genera *Aspergillus* (41%) and *Gliocladium* (26%). Active destructors were found to be the fungi of genera *Cladosporium*, *Penicillium*, *Chaetomium*, and *Fusarium* [38]. Interestingly, the taxa of plastic-degrading fungi are generally similar, regardless of the region in which the process is studied. Depending on the ecological conditions, the main group of fungi may be joined by endemic taxa specific to a particular locality or habitat. For example, while researching plastic waste in the western South Atlantic [39], scientists found many *Wallemia* isolates, an obligate xerophyte commonly found in salt water. However, the typical group of dominant fungi consists mostly of *Aspergillus* and *Cladosporium*.

One of the difficult-to-decompose synthetic polymers for fungi are polyethylenes. The stability of different grades of polyethylene depends on the molecular weight distribution of the fractions, on the presence and composition of stabilizers, etc. Recent scientific publications have confirmed the ability of microscopic fungi in natural habitats to destroy polyethylene of both high and low density. *Aspergillus oryzae* is the most active decomposer of polyethylene [35,40,41], as well as strains of the species *Penicilum oxalicum*, *P. chysogenum* [35], *P. simplicissimum* [42], and others.

The most common disposable plastic among the various synthetic plastics is polyethylene terephthalate, which is a thermoplastic polymer resin of the polyester family. This transparent, durable, and lightweight plastic is widely used for packaging, including disposable drink bottles. The fungi of the genera *Aspergillus*, *Fusarium*, and *Humicola* [43] are involved in its degradation in nature.

Other types of polymeric materials are also destroyed by fungi. Polyurethanes are a versatile class of synthetic polymers used in a variety of products in medicine, the automotive industry, and other industries. Fungi are the main destroyers of polyurethanes in nature [44]. The micromycetes *Alternaria*, *Aspergillus*, *Phoma*, *Penicilium*, *Plectosphaerella*, *Geomyces*, *Nectria*, and others have been isolated from placed in soil polyurethane specimens when the fungi have access to other sources of nutrients. *Geomyces pannorum* was shown to have the highest frequency of occurrence in this polymer decomposition method [45].

However, there are fungi that can grow in nutrition media where polyurethane is the only source of carbon. Such strains have been found in *Cladosporium cladosporioides* sensu lato [46], *Penicillium*, *Alternaria* [47], and others.

Despite the very wide range of fungi involved in the biodegradation processes of various polymer materials, it is possible to name the taxa with a high frequency of occurrence. The genera of these fungi are shown in Table 1, and the character of impact and properties of industrial materials changing under the influence of fungi are also indicated.

Both our experience and numerous scientific publications point to the conclusion that fungi capable of breaking down polymeric materials belong to several dozen genera, but the most common are representatives of the genera *Aspergillus*, *Penicillium*, and *Cladosporium*. This is usually independent of the location of the research site.

For example, when studying Antarctic fungi at sites affected by various anthropogenic impacts (soil from a petroleum leakage site, including diesel fuel, gasoline, and aviation kerosene, and soil from a location near an incinerator that operates on diesel fuel, etc.) near the Russian research Antarctic stations Druzhnaya, Progress, Molodezhnaya, and others, we noted micromycetes whose numbers increased at a certain level of pollution due to their enzymatic potential to degrade hydrocarbons. Most of them belonged to the genera *Aspergillus* and *Penicillium*. From Antarctic soil polluted with oil products we isolated a strain of the species *Penicillium restrictum* [48]. In the Arctic, when studying fungal communities at the Polar Experimental Station in the Murmansk Region, mycologists also identified representatives of this species, categorizing them as “typical frequent” in oil-contaminated soil, while in the same undisturbed soil they are categorized as “typical rare” [49].

**Table 1:** Degradation of some industrial materials by fungi.

Type of the Material	Fungal Action	Change in Properties during Biodestruction	Fungal Genera Most Actively Involved in Biodegradation	References
Fuels and lubricants, including petroleum fuels	Assimilation of hydrocarbons leading to degradation of the material	Viscosity reduction, change in acid number and oxidation stability	*Aspergillus*, *Penicillium*, *Fusarium*, *Hormoconis* (*Amorphotheca*), *Monascus*, *Neosartorya*, *Paecilomyces*, *Talaromyces*, *Graphium*, *Cunninghamella*	[14,15,25]
Lacquers and paint coatings	Oxidation, reduction, hydrolysis, esterification, decarboxylation	Change in density, color, viscosity reduction, formation of gases	*Acremonium*, *Alternaria*, *Aspergillus*, *Aureobasidium*, *Cladosporium*, *Fusarium*, *Penicillium*, *Trichoderma*, *Pullularia*	[33,34]
Polymer materials and their components	Oxidation, hydrolysis, esterification, acidification	Change in color, structure, tightness, strength		
polyurethane			*Aspergillus*, *Alternaria*, *Chaetomium*, *Cladosporium*, *Fusarium*, *Geomyces*, *Gliocladium*, *Nectria*, *Penicillium*, *Pestalotiopsis*, *Phoma*, *Trichoderma*	[45,46,47,50,51,52,53,54]
polyethylene			*Aspergillus*, *Aureobasidium*, *Cladosporium*, *Fusarium*, *Penicillium*, *Phanerochaete*	[35,40,41,42,55,56,57]
polyethyleneterephthalate			*Aspergillus*, *Fusarium*, *Humicola*	[43]
polystyrene			*Gloeophyllum striatum*, *Gloeophyllum trabeum*	[58]

## 3. Fungal Enzymes Involved in Plastic Degradation

There is evidence that biodegradation is an enzymatic process [59]. In the biodegradation process, plastics react with oxygen in the air, and then microorganisms release enzymes that decompose the plastic into simple substances, such as carbon dioxide and water [35]. The most common decomposition of plastic materials occurs in the process of co-oxydation, when, along with hard-to-reach substrates, fungi have access to other easily degradable substrates. However, there are fungi that have a pool of enzymes for the use of polymeric substances as the sole carbon source, though decomposition is slow in this case. This includes, for example, *Aspergillus niger*, which actually destroys polyurethane, but extremely slowly, so that visible signs of degradation do not appear until the 30th day [45]. 

Numerous studies shedding light on the assimilation mechanism of the ‘kerosene fungus’ *Hormoconis resinae* of n-alkanes, which are in aviation and automobile gasoline and diesel fuel, have been conducted [60]. *H. resinae* is thought to have a constitutive n-alkane oxidation system. The first step involves the entry of hydrocarbons into the cell by apparently active transport, although the mechanism of this process is not yet fully understood. Then, with the help of the enzymes alkane-monooxygenase, fatty alcoholoxygenase, and fatty aldehyde dehydrogenase, the process of alkane metabolism from hexadecane to hexadecanoic acid (palmitic acid) occurs, which, in turn, is involved in lipogenesis in fungal cells [27].

Microbial exposure to plastics can be carried out through enzymatic action, primarily of hydrolases such as ureases, proteases, and esterases. It is believed that biodegradation of polyester polyurethane may occur by hydrolysis of ester groups by esterase enzymes [61]. For example, from the biomass of *Chaetomium globosum* and *Aspergillus terreus* were isolated enzymes with esterase and urethane-hydrolase activities. The enzymes in these fungi were induced by adding liquid polyether polyurethane to the cultural medium, and it was found that a number of strains of the genera *Curvularia*, *Fusarium*, *Aureobasidium*, and *Cladosporium* can use polyurethane as their sole source of carbon. From the strain *Curvularia senegalensis* it was possible to isolate and purify an extracellular polyurethanease belonging to the esterase class, possessing high enzymatic activity [62]. *Aspergillus flavus* was also isolated from soil and found to have polyurethanolytic activity due to extracellular esterase. When it was developed on polyurethane, a 60% reduction in substrate weight was observed [63].

The endophyte *Pestalotiopsis microspora*, which is prone to horizontal gene transfer, degrades polyurethane both aerobically and in the absence of oxygen. It produces the enzyme polyurethanase, which belongs to the serine hydrolase family. The enzyme is extracellular and is synthesized by the fungus in a poor environment where polyurethane is the sole source of carbon [45].

There are studies confirming the involvement of hydrolases, such as lipases and cutinases, in the decomposition of various forms of plastic. Lipases can hydrolyse vegetable oils, triglycerides, and fatty acid methyl esters, while cutinases specifically hydrolyze ester bonds [64]. In Japan, a cutinase enzyme capable of decomposing polyethylene has been found in strains of the genus *Paraphoma*, and this enzyme is used with high efficiency in agriculture to decompose polyethylene films for soil mulching directly in the fields [65,66].

Hydrolases, such as proteases, may also be involved in the process of plastic degradation. In a study conducted on 22 strains of fungi capable of growth using polyurethane as a carbon source, it was shown with a high probability that the protease enzymatic activity was associated with plastic biodegradation [54].

Representatives of *Penicillium simplicissimum* are known to produce oxidases, such as laccase and manganese peroxidase, which decompose polyethylene [67]. *P. pinophilum* is a producer of a highly efficient specific depolymerase involved in the biodegradation of polyhydroxyalkanoates [61]. There are reports that non-ligninolytic fungi, such as *Aspergillus niger*, *Pseudogymnoascus pannorum*, and *Cunninghamella elegans*, involve the intracellular enzyme CytP450-dependent monooxygenase to degrade phenanthrene [68].

In general, it has been shown that fungal laccases and peroxidases involved in the decay of lignin by fungi are involved in the decomposition of polyethylene [69] and polyvinyl chloride, while esterases, such as cutinases and lipases, are successfully used by fungi in the biodegradation of polyethylene terephthalate and polyurethane [70].

Table 2 presents some of the enzymes found in fungi that are involved in the decomposition of plastic materials.

## 4. Selection of Strains for Testing Industrial Samples for Biostability

In most countries, including the Russian Federation, mould resistance tests are carried out regularly for various materials and products, including industrial technical products, polymer materials, and coatings. The tests are carried out according to industry standards established for various types of materials and articles. Different sets of mycelial fungi, with indications of their species affiliation, are used in these tests. Despite the different composition and specificity of the objects studied, it is possible to distinguish fungi which are involved in the process of destruction of almost all industrial materials. These are used in biostability trials.

To select the strains for these tests, the following should be considered:

The test cultures should be sufficiently versatile. They are selected from eurytopic species that are ubiquitous and occupy different ecological niches. These fungi include *Aspergillus*, *Penicillium*, *Trichoderma*, *Fusarium*, and some other genera. Additionally, To standardise the testing process and be able to compare not only the experimental and control samples, but also data from different tests, the cultures should have an active and stable sporulation, allowing a standard suspension to be obtained for the application of fungal conidia to test surfaces. A large number of basidiomycetous fungi are involved in the process of breaking down plastics. For example, fungi of the genera *Pleurotus*, *Phanerochaete*, *Trametes*, and *Bjerkandera* form highly active peroxidases and laccases [3]. However, the use of these fungi for testing of polymeric material decomposition is difficult as these fungi do not have conidial sporulation, and it is not easy to prepare a standard suspension of them. Finally, the strains must also have a powerful enzymatic system, and the ability to degrade polymeric materials.

It should be taken into account that fungi of the same species, but different strains, may present varying levels of aggression towards degradable materials. The strain-biodestructors that are used to perform tests of industrial materials for degradation resistance are stored in the VKM (Table 3). For example, the strains *Aspergillus terreus* VKM F-1025, *Aspergillus brasiliensis* (formerly known as *A. niger*) VKM F-1119, *Paecilomyces variotii* VKM F-378, and *Penicillium aurantiogriseum* (syn. *P. cyclopium*) VKM F-245 have shown high efficiency in terms of degradation of the physical and chemical properties of hybrid composites based on chitosan and acrylic polymers [82,83]. *Alternaria alternata* strain VKM F-1120 has been used for 2-4-6-trinitrotoluene decomposition [84], and the *Penicillium ochrochloron* strain VKM F-2032, isolated from fluorolone materials, was protected by a USSR patent in 1977 as a biodegradant fungus which actively grows on polymeric coatings.

Some of the strains currently in use in Russia are also being applied in other countries to assess the fungal resistance of various samples. The US Microbac Laboratory Services (http://ww.microbac.com/, accessed on 27 December 2022.) has published a list of tests, methods, and standards for testing industrial materials for resistance to fungal damage. In these tests, specific fungi from the American Type Culture Collection (ATCC) are used, and the identification numbers of the strains are included in each test. A number of the strains used are also stored in the VKM under the numbers VKM F-1119, VKM F-1115, VKM F-1117, and VKM F-1116, and are used in the Russian Governmental Standards to determine the mould resistance of industrial materials (Table 3).

International test ASTM G21-15 (standard practice for determining resistance of synthetic polymeric material to fungi) is a qualitative test that uses high concentrations of conidia from five different fungi species to determine the resistance of synthetic polymeric materials to fungal growth. The five strains of *Aspergillus brasiliensis* (formerly known as *A. niger*), *Aureobasidium pullulans*, *Chaetomium globosum*, *Trichoderma virens* (formerly known as *T. viride*), and *Penicillium funiculosum* (formerly known as *P. pinophilum*) are used as test cultures here. Approximately the same set of cultures is used in the International Standard ISO 846: 2019—(Plastics, Evaluation of the Action of Microorganisms). Method A of this test involves testing plastics for mould resistance. The difference between the two tests is that the latter uses the *Aureobasidium pullulans* strain instead of the *Paecilomyces variotii* strain.

In similar Russian tests in the study of biodegradation of polymeric materials and their components, nine cultures of fungi are being used. In addition, the strains of species *Aspergillus sojae* (formerly known as *A. oryzae*), *Aspergillus terreus*, *Penicillium aurantiogriseum*, and *P. chrysogenum* are being utilised. This is because the representatives of these species are active, and they are often the main biodestructors of polymeric composite materials [85,86,87].

The fund of VKM fungi-biodestructors is constantly being updated in the hopes of further development of methodological recommendations for assessing the biostability of materials.

Thus, the active role of the micromycetes *Clonostachys rosea* var. *catenulata* VKM F-3955, *C. solani* VKM F-3964, *Thrichoderma harzianum* VKM F-3962, and others in the biodegradation of polymers, particularly polyvinyl alcohol, polyurethane, and latexes based on acrylic acid used in construction as paintwork materials, has been shown in scientific studies [88]. Seventeen strains of soil micromycetes that are polymer biodestructors were accepted into the VKM collection based on the results of these studies.

The collection was enriched with a unique strain of *Monascus floridanus* (VKM F-4444), already mentioned as a ‘kerosene fungus’, which grows rapidly in aviation fuel and decomposes it at a high rate.

**Figure 1:**
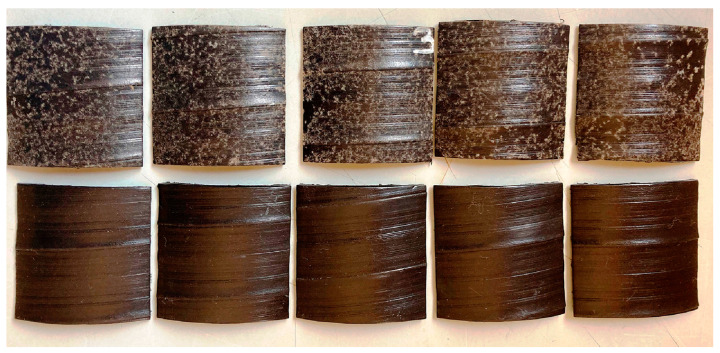
Testing of the fungal resistance of paint and varnish coatings (bottom row-control). After 28 days of testing (temperature 30 °C, relative humidity more than 90%) on the surface of the material the growth of test-cultures in the form of a light-colored plaque, in particular *Aspergillus terreus* (VKM F-1025) is visible with the naked eye.

**Figure 2:**
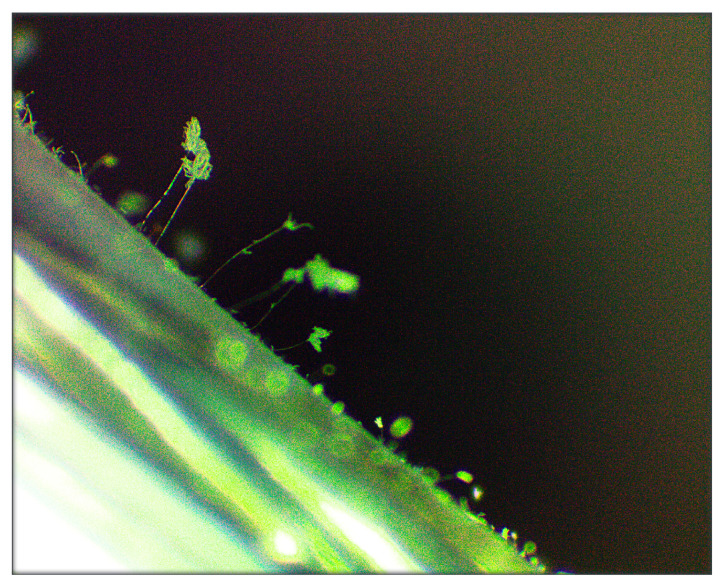
Sporulation of test-culture *Paecilomyces variotii* VKM F-378 on the surface of the testing specimen of polyethylene film coating.

In the last five years, confirmation of the biological activity of a set of strains used in the All-Russian Collection of Microorganisms (VKM) was achieved with 663 samples of various polymer materials (mainly protective polyethylene and lacquer coatings) and products. It was found that the polymer composition influences resistance to biodamage; most of the investigated materials were resistant to the influence of fungi, but materials without such properties (unpublished data) have been also identified. Figure 1 shows the development of test cultures 28 days after the application of fungal spores on the polymer surface. In Figure 2, sporulation of a culture of *Paecilomyces variotii* VKM F-378 on a film of polyethylene coating is clearly visible. The results of VKM test cultures used in the development of biodegradable materials are presented below.

## 5. Application of Test Micromycetes from the VKM Collection for Investigations in the Field of Biodegradable Material Development

Another research field using mould fungi for testing is in the development of biodegradable polymer materials. One of the mandatory stages in the study of such materials is an exploration of their biodegradability. Tests of fungi resistance allow this ability to be unambiguously established.

In recent decades, interest in the creation of biodegradable polymer materials has increased due to the urgency of solving the problem of accumulation and disposal of polymeric wastes. Leading researchers around the world are developing more and more new formulations that could replace products made from synthetic and nondegradable, primarily oil-based polymers, for example, [89,90,91]. Such materials can be used as packaging containers, disposable tableware, mulching films, etc. One of the mandatory stages in the study of biodegradable materials is an exploration of their biodegradability. Previously, numerous authors have shown that this characteristic is essential in describing the properties of the obtained biodegradable compositions. It is therefore important to comprehensively investigate biodegradability using different methods, including tests of mould resistance using test cultures of polymers from VKM, assessing the mass loss of samples after exposure in soil for a certain period of time, fourier-transform infrared spectroscopy (FTIR), evaluating the change in structure by scanning electron microscopy (SEM), and so forth. 

In order to be able to compare the results with other research, all the fungal resistance tests were carried out using a standard set of test organisms, consisting of nine strains used in the study of degradation of polymeric materials (Table 3), namely *Chaetomium globosum* VKM F-109, *Penicillium chrysogenum* VKM F-245, *Penicillium aurantiogriseum* VKM F-265, *Paecilomyces variotii* VKM F-378, *Aspergillus terreus* VKM F-1025, *Penicillium pinophilum* VKM F-1115, *Trichoderma virens* VKM F-1117, *Aspergillus brasiliensis* VKM F-1119, and *Aspergillus sojae* VKM F-2096. All tests were performed under standard conditions: the temperature was 30 °C, the relative humidity was over 90%, and the incubation time was up to 84 days.

In earlier studies [92,93,94], the authors obtained compositions based on low-density polyethylene (LDPE) with various polysaccharides (cellulose, ethyl cellulose, starch, chitin, chitosan) under conditions of shear deformation. The development of such compositions, allowing one to utilize large-tonnage synthetic polymers, led to a combination of the properties of both components; namely, good mechanical properties along with biodegradability. It has been shown that fungal resistance depends on the nature of the polysaccharide used and its availability in the composition. Compositions based on LDPE with starch and chitin [92] show minimal fungal resistance, while the introduction of poly(ethylene oxide) [93,94] and the creation of LDPE compositions with two different polysaccharides [95,96,97] intensified the growth of fungi. According to the existing system for assessing the intensity of their growth (six-point scale), these compositions received the maximum five points, as more than 25% of their surface was covered with fungal mycelium, which could be seen with the naked eye.

In further works, the authors developed compositions based on various polymers of natural origin (polysaccharides and polylactide (PLA)). Thus, in [97,98,99,100], the authors showed it is possible to obtain compositions based on PLA with various polysaccharides under the conditions of shear deformation and studied their properties, including biodegradability. PLA decomposes only under severe conditions (compost, sea water, etc.). However, the PLA-based compositions with polysaccharides exhibited low fungal resistance. In the study of PLA compositions with microcrystalline cellulose and ternary systems with poly(ethylene glycol) (PEG) [97], it was possible to identify fungal species that exhibited the maximum growth rate. Thus, after 21 days of testing, *Aspergilus* fungi were dominant, while on the 45th day of testing, conidial structures characteristic of *Penicillium* fungi were already clearly distinguishable. Rogovina et al. [98] showed that biodegradation proceeds most intensively in samples containing starch and PEG. Moreover, it was shown that after 84 days of testing in the case of a binary composition, almost 100% of the surface was covered with a dark grey layer caused by *Aspergillus brasiliensis* (VKM F-1119), while for the system with PEG, sample pigmentation indicated the dominant action of *Aspergillus terreus* (VKM F-1025).

Recent studies have been aimed at creating compositions based on LDPE, PLA, and starch [99,100]. According to tests of the fungal resistance of these systems, the same pattern was revealed; in compositions with a high content of polysaccharide starch, the biodegradation process proceeded more intensively (i.e., minimal fungal resistance was exhibited). More than 25% of the sample surface was covered by fungal mycelium that could be visualized by the naked eye.

Thus, studies conducted with the standard set of test cultures have made it possible to detect the patterns of destruction of various polymer compositions.

## 6. Conclusions

The creation of biodegradable polymer materials, as well as of protective compositions to reduce biodegradation of industrial products during operation, requires effective testing of materials for resistance to biodamage. The degree and nature of changes in the properties of the tested samples can be determined by the qualitative and quantitative composition of the metabolites of the microorganisms involved in the biodegradation process. Fungi have an important role in biodegradation processes, which is reflected in the creation of different tests governing the assessment process for mould resistance. A specific set of test cultures allows standardised research and, as a consequence, the ability to compare the results of studies of the fungal resistance of different materials. These fungi should have a constantly pronounced activity (aggressiveness) and be able to damage certain materials. The VKM is a member of the World Federation of Culture Collections (WFCC) and the largest collection of fungi in the Russian Federation and is, which preserves test-cultures and provides them to interested parties. Its capabilities allow us to ensure not only the viability but also the activity of the test cultures over a long period of time using modern preservation methods.

The VKM also constantly updates the list of active cultures with strains isolated directly from the foci of bio-damage materials and products. Changes in test cultures’ names related to the development of fungal taxonomy, data on the storage of these cultures in world collections, as well as the information of use of specific strains as research subject, are regularly monitored in VKM.

## Figures and Tables

**Table 2:** Fungal enzymes involved in plastic degradation.

Enzymes	Fungi	References
Catalase	*Aspergillus clavatus*	[71]
Cutinase	*Aspergillus oryzae*, *Fusarium oxysporum*, *F. solani*, *Humicola insolens*, *Penicillium funiculosum*, *Paraphoma* sp., *Trichoderma reesei*	[43,65,72,73,74]
Laccase	*Trichoderma harzianum*, *Penicillium simplicissimum*, *Aspergillus flavus*	[67,69,70]
Lipase	*Thermomyces lanuginosus*, *Rhizopus arrhizus* (syn. *R. delemar*, *R. nivea*, *R. oryzae*)	[75,76,77]
Manganese peroxidase	*Penicillium simplicissimum*, *Phanerochaete chrysosporium*, *Trichoderma harzianum*	[67,69,72,78,79]
Polyurethanase	*Curvularia senegalensis*	[62,75]
Protease	*Alternaria solani, Tritirachium album*	[75,80,81]
Serine hydrolase	*Pestalotiopsis microspora*	[45]
Urease	*Trichoderma* sp.	[53,56]

**Table 3:** List of strains used in mold resistance tests in VKM.

Number VKM F-	Species	Numbers of Strain in Other Collections of WFCC *	Type of Testing Samples					
			Technical Products	Polymeric Materials and Their Components	Fabrics, Including Synthetic Fibres	Oils and Lubricants	Paint and Varnish Coatings	Petroleum Fuels
109	*Chaetomium globosum*	LCP 679		+	+			
136	*Fusarium fujikuroi* (*F. proliferatum*)	ATCC 12616; BRL 917; CBS 183.29; DSM 893; IMI 58290					+	
234	*Penicillium brevicompactum*	no					+	
245	*Penicillium chrysogenum*	no		+	+	+	+	
265	*Penicillium aurantiogriseum* (syn. *P. cyclopium*)	ATCC 8731; ATHUM 2888; CBS 114.74; CECT 2264; DSM 1250; FRR 1888; IMI 089372; MUCL 15613; NRRL 1888		+		+		
378	*Paecilomyces variotii*	no	+	+	+	+		
406	*Scopulariopsis brevicaulis*	no	+			+		
1025	*Aspergillus terreus*	no	+	+	+		+	
1115	*Penicillium pinophilum* (*P. funiculosum*)	ATCC 9644; CBS 170.60; CCRC 31621; DSM 1960; IFO 6345; NRRL A-5245; QM 391	+	+	+		+	
1116	*Aureobasidium pullulans*	ATCC 9348; CBS 621.80; CCRC 31981; DSM 2404; IMI 145194; NCIM 1049; QM 3090	+					
1117	*Trichoderma virens* (*T. viride)*	ATCC 9645; CBS 430.54; IAM 5061; IFO 6355; IMI 45 553; NRRL 2314; QM 365	+	+	+		+	
1119	*Aspergillus brasiliensis* (*A. niger*)	ATCC 9642; CBS 246.65; CCRC 31512; DSM 63263; FERM S-2; IFO 6342; IMI 91855; NRRL A-3536	+	+	+	+		
1120	*Alternaria alternata*	no					+	
1701	*Hormoconis resinae* (syn. *Cladosporium resinae*)	no						+
2032	*Penicillium ochrochloron*	no	+		+		+	
2039	*Aspergillus niger*	ATCC 6275; CBS 769.97; CCRC 32073; CECT 2807; IFO 6341; DSM 1957; IMI 45551; NRRL 334					+	
2096	*Aspergillus sojae* (*A. oryzae*)	ATCC 14895; CBS 134.52; CCRC 30230; NRRL 1989		+				

* WFCC—World Federation of Culture Collection.

## Data Availability

Not applicable.
